# Assessment of the sanitary quality of ready to eat sesame, a low moisture street food from Burkina Faso

**DOI:** 10.1186/s12866-021-02269-0

**Published:** 2021-07-08

**Authors:** Muller K. A. Compaoré, Bazoin Sylvain Raoul Bazie, Marguerite E. M. Nikiema, Virginie Marie Dakené, René Dembélé, Dissinviel Stéphane Kpoda, Elie Kabré, Nicolas Barro

**Affiliations:** 1 Laboratoire de Biologie moléculaire, d’Epidémiologie et de Surveillance des agents Transmissibles par les Aliments (LaBESTA), Centre de Recherche en Sciences Biologiques Alimentaires et Nutritionnelles (CRSBAN), École Doctorale Sciences et Technologies, Université Joseph KI- ZERBO, 03 BP 7021, Ouagadougou 03, Burkina Faso; 2 Laboratoire National de Santé Publique (LNSP), 09 BP 24, Ouagadougou 09, Burkina Faso; 3 Unité de Formation et de Recherche en Sciences Appliquées et Technologies (UFR/SAT), Université de Dédougou, BP 176, Dédougou, Burkina Faso; 4 Université Joseph KI- ZERBO, Centre Université de Ziniaré, 03 BP 7021, Ouagadougou, 03 Burkina Faso; 5 Laboratoire de Biochimie, Biotechnologie, Technologie Alimentaire et Nutrition (LABIOTAN), Université Joseph KI-ZERBO, 03 BP 7021, Ouagadougou 03, Burkina Faso

**Keywords:** Sanitary quality, *Sesamum indicum*, Microorganisms, Low moisture food, Burkina Faso

## Abstract

**Background:**

Microbial contamination of edible low moisture food poses a significant public health risk for human. In this study, the microbial quality of sweet dehulled sesame seed croquettes, salted dehulled sesame seed and the raw sesame seed, sold under ambient conditions were examined. The samples were collected in the cities of Burkina Faso. The first type is sweet dehulled sesame seed croquettes (n_1_ = 25); the second type is salted dehulled sesame seed (n_2_ = 25) and the third type is raw sesame seed (n_3_ = 25). Assessment of the microbial quality was based on the total aerobic mesophilic bacteria, the thermotolerant coliforms, the yeasts and moulds, the *E. coli,* and the *Salmonella* spp. using ISO methods.

**Results:**

The results showed the presence of microorganisms varying from <1.0 to 1.72 × 10^5^ CFU g^− 1^ for thermotolerant coliforms, from <1.0 to 6,12 × 10^6^ CFU g^− 1^ for the total mesophilic aerobic flora and from <1.0 to 8.10 × 10^5^ CFU g^− 1^ for yeasts and moulds. The higher contaminations rates were mostly observed in raw sesame seed samples. No *E coli* or *Salmonella* pathogens were detected. Based on international standards of dehydrated food, 50.67% of the ready to eat sesame are satisficing while 17.33% are acceptable and 32% are not satisficing.

**Conclusion:**

Attention should be emphasized on the processing practices, especially in crowded places where RTE sesames seeds are mostly sold. The high numbers of all microbial groups in these sesame seed samples suggested that the production of RTE sesame seed should be improved by better hygiene. This study highlights also that RTE sesame seed might harbor a wide range of microorganisms when processes are weak of hygiene.

## Background

**Fig. 1:**
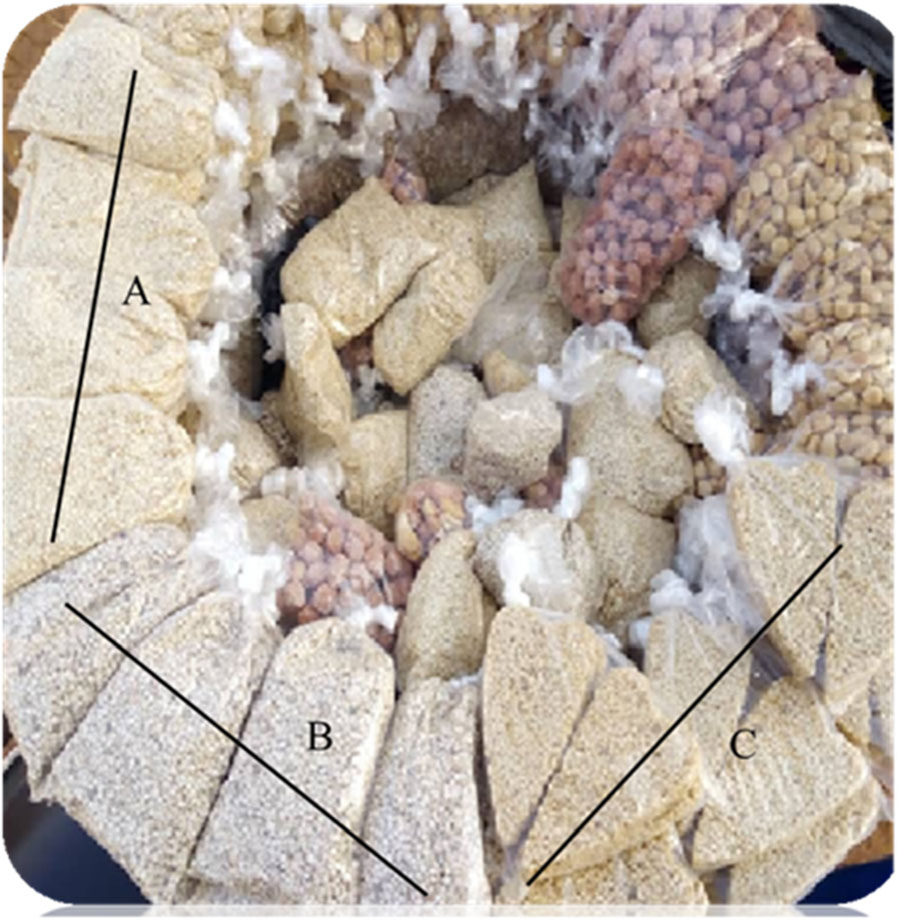
Street vendor set: A = Salted dehulled sesame seed croquettes; B = Raw sesame seed; C = Sweet dehulled sesame seed. Black line indicated the limit of each sample

RTE (Ready-To-Eat) food are those that do not require any further processing (such as cooking) prior to consumption by the consumer [1]. RTE such as sesame seed is a very popular low-moisture food, sold almost everywhere by ambulant vendors in all regions in Burkina Faso (Fig. 1). It is customary known as a small gift for children when parents are travelling within the country. Low-moisture foods were once thought to be relatively safe with respect to foodborne illness risk since low water activities do not permit the growth of foodborne microorganisms [2]. Recently over the years, a number of incidences related to outbreaks caused by the presence of food-borne pathogens in low water activity (a_w_) foods have increased [3, 4]. It is thought that some pathogens can survive in low-moisture foods but do not grow in them until moisture is deliberately added such as during further preparation and storage or accidently exposed such as water. According to [4] foods with a_w_ < 0.85 are considered as low moisture food and include cereals and many other dry food as grains and seeds (e.g., sesame, melon, pumpkin, linseed).

Sesame seed processing is still at the traditional stage in Burkina Faso. It is run by artisanal and semi-artisanal processors who are individuals, companies, associations and some pastries [5]. These processed products are generally sweet dehulled sesame croquettes, salted dehulled sesame seeds and raw sesame seeds. Sesame croquettes are sweet RTE food shaped differently after caramelization in sugar. According to the International Marketing – Management Consulting Group [6], sesame seed is consumed in the raw state (26%), in cookies form (35%), as ingredients for cooking (25%), as oil (6%) and as pastry ingredient (6%). Although, the majority of the national sesame production is export oriented, there is few data about the quality of processed sesame seed for local consumption. Hygiene conditions are poor when foods are produced in non-industrial establishments, mainly due to the fact that the necessary infrastructure for technologically adequate processes is not available [7]. Additives are sometimes used to enhance the food taste and conservation or to lowered microbial growth. Sugar either salt decreases a_w_ of the product thereby inhibiting microbial growth [8]. Sesame seeds are usually dehulled and roasted before use; this improves their functional properties, releases more flavor and color, and improves their sensorial quality [9]. Also, dehulling removes relatively high amounts of antinutritional oxalic acid and fiber contained in the testa (seed coat), resulting in lighter-colored, less bitter-tasting seeds [9]. From these dehulled sesames seeds, the products are obtained by caramelization in sugar or by soaking the seeds in salty water before roasting. These processes are usually done manually. Published papers highlighted that there is potential health risk associated with initial contamination of foods by pathogenic bacteria as well as subsequent contamination by vendors during preparation and through post-cooking handing and cross contamination [10, 11]. The purpose of this study was to assess the microbiological quality of three types of edible sesame seed sold in the street of fourteen (14) provinces. The microorganisms of interest were those that can decrease the quality of sesame seed products and cause public health issue and those that can withstand high concentration of sugar and salt. These microorganisms include thermotolerants coliforms, Salmonellas, *E. coli* and yeasts and moulds.

## Methods

### Samples collection and storage

**Fig. 2:**
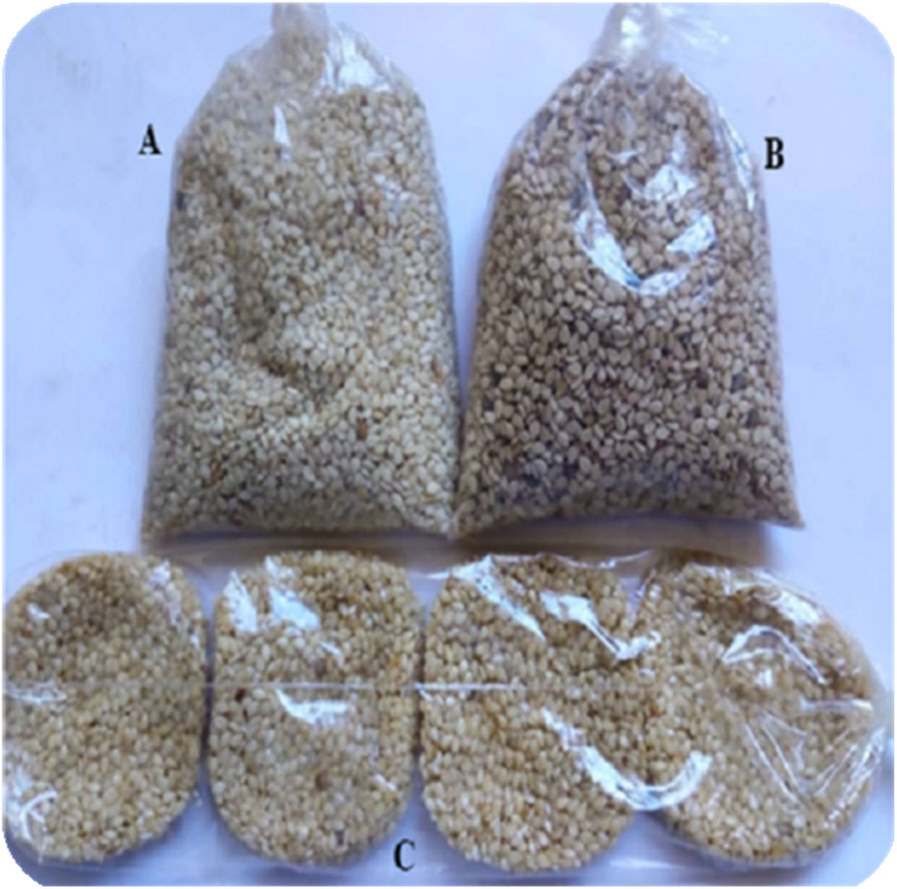
The three different categories of processed edible sesame (A = Salted dehulled sesame; B = Raw sesame; C = Sweet dehulled sesame)

**Fig. 3:**
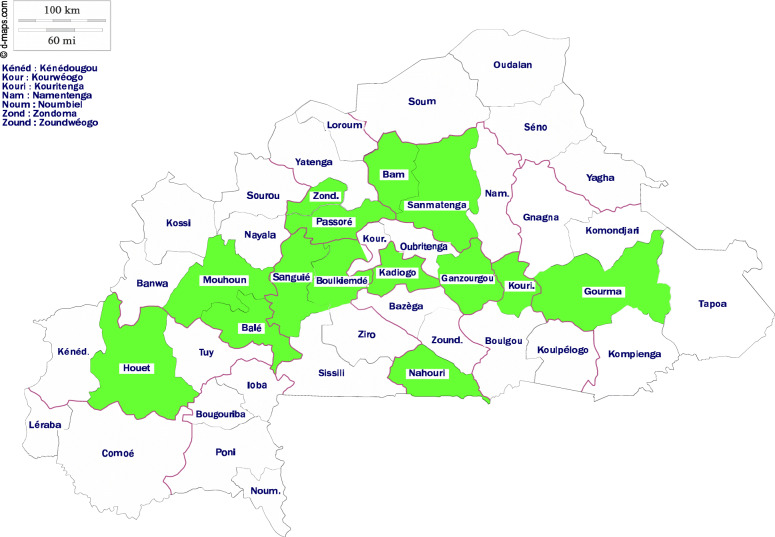
Source: https://www.d-maps.com/carte.php?num_car=25733&lang=fr

Samples of three different categories of processed edible sesame seed (Fig. 2) were collected in some crowded cities, roads and major streets of Burkina Faso. Sampling was carried out in duplicate according to the production batches in fourteen (14) provinces (Fig. 3). Sample were transferred aseptically in sterile plastic bags, sealed and stored at room temperature prior for analyses. Sampling of salted sesame seed and raw sesame seed was carried out in duplicate according to the production batches and then vigorously mixed to obtain a homogeneous sample of at least 50 g. Sweet dehulled sesame seed croquettes was also sampled in duplicate according to the production batches and then crashed in sterile bag with Bag Mixer, Interscience France for 2 min to obtain a homogeneous sample of at least 50 g. The following samples were collected:

- Sweet dehulled sesame seed croquettes (n_1_ = 25);
- Salted dehulled sesame seed (n_2_ = 25) and CDC
- Raw sesame seed (n_3_ = 25)

**Fig. 4:**
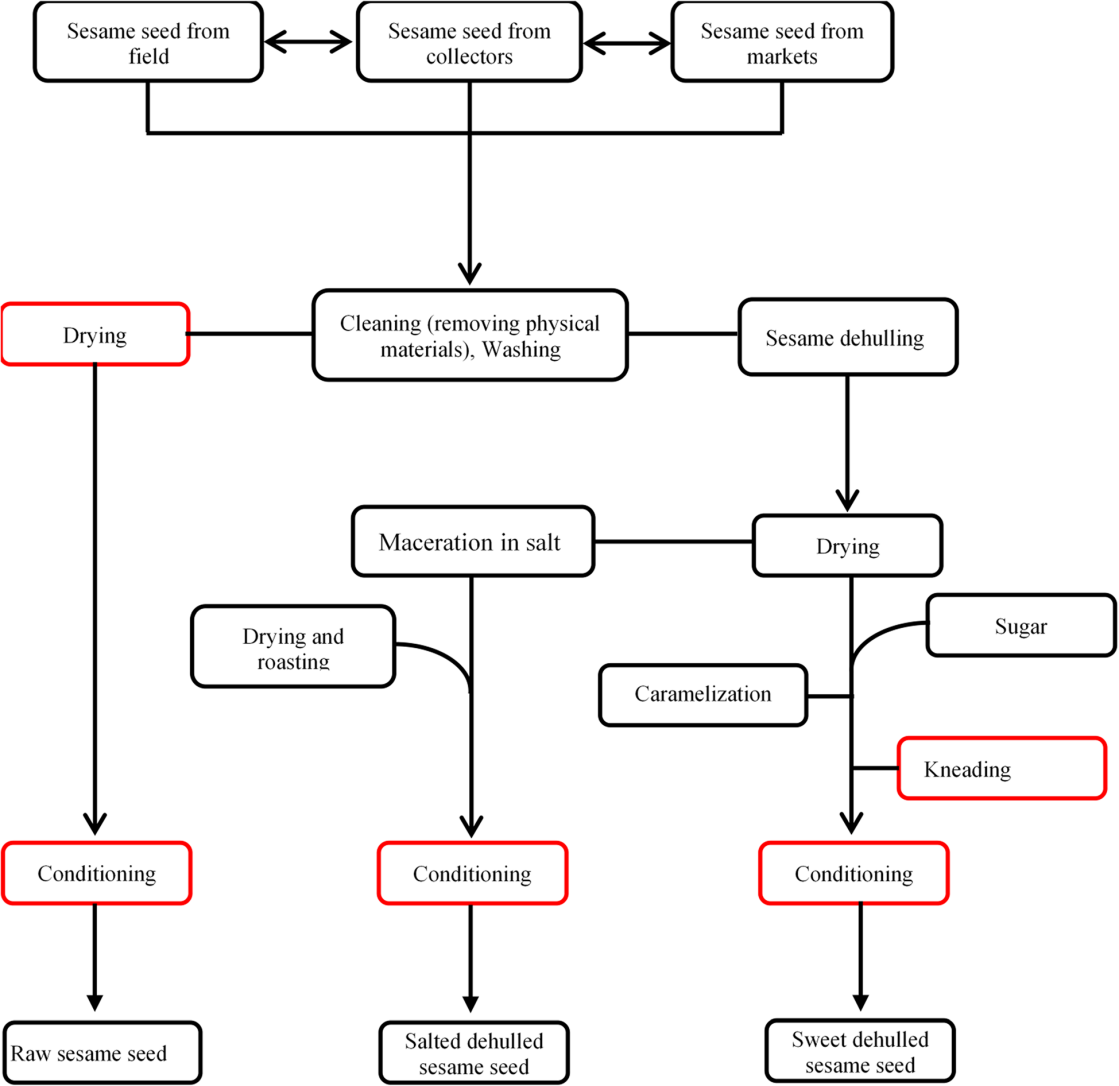
RTE sesame processing flowchart. Red color indicates critical points

The processing steps and parameters for the three types of the RTE sesame were obtained through a ten-minute interview with sesame processors in order to establish the production flowchart (Fig. 4). The set of processes used to obtain the three types of sesame seeds includes a common step, namely cleaning and washing (removal of physical matter). From this step, the raw RTE sesame seeds were prepared by drying the seeds with local dryers (temperature > 70 °C until total dried) and packaging them in plastic bags which quality are doubtful (might not be sterile). The sesame was dehulled by traditional processes which consisted in pounding it manually in a mortar. The dehulled salted sesame seeds were obtain by a 30 min maceration in about 10% salted solution at ambient temperature. The salt contributes to give taste to the sesame seed but also to optimize the conservation [8]. Seeds was then been dried lightly, roasted (temperature > 100 °C until total dried) and packaged in same kind of plastic bags at ambient conditions.

Concerning sweet dehulled sesame seeds croquettes, the transformation processes were identical to that of salted sesame with the difference that the dehulled sesame have been caramelized (temperature > 100 °C) in up to 20% sugar. Before total cooling, the caramelized sesame undergoes kneading at ambient temperature in order to give it different shapes (circles, heart, lozenge ...). The final product was then packed in a plastic bag usually at ambient conditions. Figure 4 summarizes all the transformation processes involved in obtaining the three types of RTE sesame.

### Microbial analysis of the samples

Twenty-five (25) grams of each sesame seed and sesame croquettes samples were homogenized into 225 mL of sterile buffered peptone water (Liofilchem diagnostic, Italy). Further tenfold serial dilutions were made with in sterile buffered peptone water. Duplicate plates were made for each sample at each dilution under ISO 6887 standard methods. Microbial counts were expressed as colony-forming units per gram of sesame (CFU g^− 1^)

- *Total aerobic mesophilic bacteria*, were counted among all the sesame samples onto standard plate count (PCA) agar (Conda Pronadisa, Spain) under NF ISO 4833: 2003. Plates were incubated at 30 ± 1 °C for 72 h. After incubation the number of colonies was counted on the plate with less than 300 colonies.
- *Thermotolerant coliforms* known to be an indicator of fecal contamination were counted onto standard violet red bile lactose (VRBL) agar (Conda Pronadisa, Spain) incubated at 44.5 ± 0.5 °C for 24 to 48 h under NF V08–017:1980. Only the Petri dish containing less than 150 colonies were considered.
- *Escherichia coli* was identified through the IMViC test from thermotolerant coliforms.

Suspected colonies were selected and subcultured on Nutrient Agar at 37 °C for 24 h. Pure cultures grown on Nutrient Agar were used for Oxidase test and determination of IMViC pattern (indole, methyl red, Voges Proskauer and citrate utilization test) following Standard Procedures for food Analysis. Positive clones were transferred into Levine BBL™ Eosin Methylene Blue Agar (EMB) Agar France, which was incubated at 37 ± 1 °C for 24 h. *Escherichia coli* ATCC 8739 was uses as positive control for all samples.

- *Salmonella spp.* was investigated according to the standard - Horizontal method for detection of *Salmonella* spp. ISO 6579:2007. Briefly, the non-selective enrichment was done by adding 25 g of each sesame sample into 225 mL buffered peptone water incubated at 37 °C for 18 to 20 h. The selective enrichment step was performed onto both tetrathionate (Müller-Kauffman) (Liofilchem diagnostic, Italy) and Rappaport Vassiliadis Soy (Difco laboratories) broths incubated respectively at 37 ± 1 °C and 42 ± 1 °C for 18 to 20 h. A brilliant green at 0.95% was added to the selective media Tetrathionate broth in order to inhibit the growth of Gram-positive bacteria. Isolations were performed onto Xylose Lysine Deoxycholate (HiMedia Laboaratories, India) and *Salmonella-Shigella* (HiMedia Laboaratories, India) agars. Suspected colonies were purified on nutrient agar and then submitted to API 20E (BioMérieux) test for biochemical confirmation. *Salmonella typhimurium* (ATCC 14028) and *Salmonella enteritidis* (ATCC 13076) were used as positive control. The Key biochemical tests including the fermentation of glucose, negative urease reaction, lysine decarboxylase, negative indole test, H2S production, and fermentation of dulcitol [12].
- *Yeasts and moulds* were counted onto standard yeast extract glucose chloramphenicol (YGC) agar (HiMedia Laboaratories, India) incubated at 25 ± 1 °C for 5 days following ISO 7954:1988. The growth of moulds was checked every day in order to avoid invading colonies. Considered Petri dish for bacterial counting were less than 150 colonies.

### Statistical analysis

Statistical analysis was done using the Epi Info Version 7.2.2.6 (Centers for Disease Control and Prevention [CDC], Atlanta). Multivariable logistic regression was used to estimate odds ratios (ORs) with 95% confidence intervals (95% CI) also calculated. Comparison tests were done to determine whenever there were significant differences (*P* ≤ 0.05) within these types of sesame seeds and sesame croquettes samples. Values obtained in the counts were transformed to decimal logs.

### Criteria of appreciation

There are no Burkinabe standards to assess the microbial quality of processed sesame seeds and sesame croquettes. Therefore, the criteria assigned to dehydrated plant products formulated by [13] have been used. The criteria decreed by the government of Québec concerning nuts, dried fruits, fruit powder, nut paste have been also considered [14] (Table 1).

**Table 1:** Microbiological counts of three types of ready to eat sesame (log10 CFU g^− 1^)

	*Thermotolerant coliforms*			*Total aerobic mesophilic bacteria*			*Yeasts and moulds*			*Salmonellas* spp	*E. coli*
	***Counts***			***Counts***			***Counts***			***Prsc/Abs/25 g***	***Prsc/Absg***
Samples	DW	DS	RS	DW	DS	RS	DW	DS	RS	DW, DS, RS	DW, DS, RS
**1**	<1.0	<1.0	3.97	3.18	3.70	5.11	2.00	**2.48**^**a**^	3.79	Abs	Abs
**2**	<1.0	<1.0	<1.0	3.00	3.30	3.93	<1.0	2.00	2.85	Abs	Abs
**3**	<1.0	<1.0	<1.0	3.90	<1.0	3.74	2.85	<1.0	2.48	Abs	Abs
**4**	4.93	<1.0	4.93	5.18	3.30	5.03	3.91	<1.0	3.73	Abs	Abs
**5**	<1.0	<1.0	3.23	3.00	<1.0	4.37	<1.0	<1.0	2.85	Abs	Abs
**6**	3.30	<1.0	3.26	3.93	3.18	3.98	<1.0	<1.0	2.85	Abs	Abs
**7**	2.00	**2.30**^**a**^	<1.0	3.18	3.30	4.31	<1.0	<1.0	2.90	Abs	Abs
**8**	2.48	<1.0	3.92	2.48	<1.0	4.51	2.00	<1.0	2.90	Abs	Abs
**9**	3.51	2.02	4.39	3.67	3.30	5.79	2.41	2.00	3.89	Abs	Abs
**10**	3.42	1.90	**5.24**^**a**^	3.55	2.00	**6.52**^**a**^	2.30	<1.0	**5.91**^**a**^	Abs	Abs
**11**	<1.0	1.60	3.71	2.48	2.48	3.93	<1.0	2.00	3.41	Abs	Abs
**12**	<1.0	<1.0	4.51	2.95	3.16	5.23	1.18	1.00	3.39	Abs	Abs
**13**	<1.0	<1.0	4.60	3.29	3.66	5.13	1.40	<1.0	4.55	Abs	Abs
**14**	**4.54**^**a**^	<1.0	<1.0	**6.79**^**a**^	4.37	6.01	**3.98**^**a**^	1.65	3.03	Abs	Abs
**15**	<1.0	<1.0	3.23	2.78	3.48	3.88	1.00	<1.0	2.56	Abs	Abs
**16**	<1.0	<1.0	3.28	2.98	**4.77**^**a**^	3.45	<1.0	1.48	1.78	Abs	Abs
**17**	2.77	<1.0	3.58	4.72	3.15	5.12	0.70	2.26	4.10	Abs	Abs
**18**	<1.0	<1.0	1.90	3.79	<1.0	3.85	0.70	<1.0	2.37	Abs	Abs
**19**	<1.0	<1.0	<1.0	3.45	<1.0	3.46	1.88	<1.0	2.39	Abs	Abs
**20**	<1.0	<1.0	1.00	4.11	<1.0	3.00	1.74	<1.0	1.70	Abs	Abs
**21**	<1.0	<1.0	4.86	2.80	1.70	5.16	<1.0	<1.0	2.65	Abs	Abs
**22**	3.83	<1.0	<1.0	5.53	2.45	4.54	1.18	1.18	2.51	Abs	Abs
**23**	<1.0	<1.0	<1.0	4.35	3.52	4.85	1.18	2.06	2.45	Abs	Abs
**24**	3.79	<1.0	<1.0	4.95	2.11	4.00	1.48	<1.0	2.00	Abs	Abs
**25**	4.44	<1.0	2.61	4.76	1.48	5.34	1.00	<1.0	2.48	Abs	Abs
Average	3.83	1.23	4.26	4.74	3.65	5.39	2.89	1.6	4.55	Abs	Abs

a = maximum value in each sample group; minimum value = <1.0; Prcs: presence Abs: absence

## Results

The enumeration (log_10_ CFU g^− 1^) of microorganisms such as total flora, thermotolerants coliforms, yeast and mould as well as the presence or absence of *Salmonella* and *E. coli* obtained following assessment of 75 sesame seeds and sesame croquettes samples are shown in the Table 1.

The results showed the presence of microorganisms independently of the sample nature. Thermotolerant coliforms were found in samples as follow: 44% in sweet dehulled sesame croquettes, 16% in salted dehulled sesame seed and 68% in raw sesame seed. The means concentration was 6.79 × 10^3^; 1.70 × 10; 1.84 × 10^4^ CFU g^− 1^ (respectively for sweet dehulled sesame croquettes, salted dehulled sesame seed and raw sesame seed). The thermotolerant coliforms column independently of the sample type, showed that 43 samples (57.3%) out of 75 sesame samples had no coliforms ((95% CI: 45.4–68.7). The majority of these samples belong to the salted dehulled sesame seed (28%), followed by Sweet dehulled sesame croquettes (18.67%) and raw sesame seed (10.66%).

92% of the samples have shown a presence of microorganisms. The total aerobic mesophilic flora varies from 3 × 10 to 6.12 × 10^5^ CFU g^− 1^. The means concentrations of the total aerobic mesophilic flora were 5.5 × 10^4^; 4.5 × 10^3^; 2.5 × 10^5^ CFU g^− 1^ respectively for sweet dehulled sesame croquettes, salted dehulled sesame seed and raw sesame seed. All the six samples (8, 95% CI: 3–16.6) which did not show any microorganisms in the total aerobic mesophilic flora belong to the salted shelled sesame group. Therefore, concerning the flora, the two other types of sesame seed samples showed the presence of microorganisms in all the analyzed samples with high loads in the raw sesame seed samples.

The total aerobic mesophilic flora has the highest number of microorganisms in these three types of sesame seed samples as compared to the thermotolerant coliforms and the yeasts and moulds.

### Pathogens detection

Although thermotolerant coliforms were detected in 32 samples (42.7%), *Escherichia coli* neither *Salmonella* spp., were detected in any of the 75 analyzed samples.

### Yeast and moulds

**Fig. 5:**
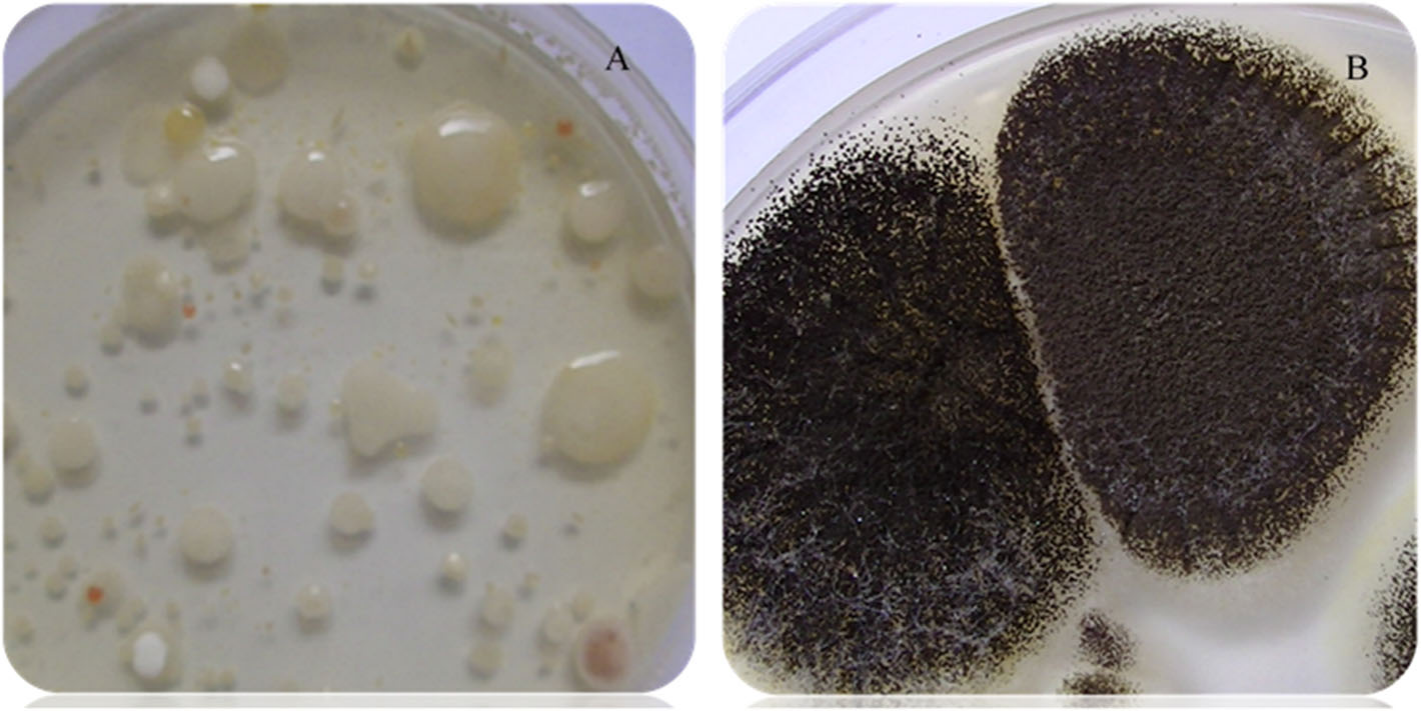
Predominant morphologies of yeast and mold encountered. 5A: yeast colonies; 5B: molds colonies

Concerning the yeast and mould, 22 samples (29.3, 95% CI: 19.4–40.9) out of the 75 did not showed any microorganisms whose 15 (20%) belong to salted dehulled sesame seed and 7 (9.3%) to sweet dehulled sesame croquettes. All the raw sesame seed sample gave at least 10 CFU g^− 1^ yeasts and moulds. The means concentrations in this group were 7.7 × 10^2^; 4.0 × 10; 3.6 × 10^4^ CFU g^− 1^ respectively for sweet dehulled sesame croquettes, salted dehulled sesame seed and raw sesame seed. Different morphologies of yeast and mold were observed as showed in Fig. 5.

The maximum loads in thermotolerant coliforms, in total flora and in yeasts and molds were observed with the same sample of Sweet dehulled sesame croquettes and raw sesame seed. In the case of salted dehulled sesame seed, three different samples carried these maximum values.

## Discussion

Food quality has always been a concern for human being especially in developing countries. The results of the present study showed that the three types of RTE sesame seed (sweet dehulled sesame croquettes, salted dehulled sesame seed and raw sesame seed) harbor microorganisms such as total flora, thermotolerant coliforms and yeasts and moulds up to 72% in sesame sweet dehulled sesame croquettes, 44% in salted dehulled sesame seed and 89.33% in raw sesame seed.

Raw sesame seed had the highest counts of thermotolerants coliforms, total aerobic mesophilic bacteria and yeasts and moulds as compared to dehulled sweet and dehulled salted sesame. The means were 4.26 log_10_UFC.g^− 1^; 5.39 log_10_UFC.g^− 1^ and 4.55 log_10_UFC.g^− 1^ respectively for thermotolerants coliforms, total aerobic mesophilic bacteria and yeast and mould. Raw sesame seed was followed by sweet dehulled sesame croquettes on which the means of thermotolerants coliforms, total aerobic mesophilic bacteria and yeast and mould were respectively 3.83 log_10_UFC.g^− 1^; 4.74 log_10_UFC.g^− 1^ and 2.89 log_10_UFC.g^− 1^. The lower contaminated was salted dehulled sesame seed with the average of 1.23 log_10_UFC.g^− 1^; 3.65 log_10_UFC.g^− 1^ and 1.6 log_10_UFC.g^− 1^ respectively for thermotolerants coliforms, total aerobic mesophilic bacteria and yeasts and moulds. Based on recommended microbiological criteria for dehydrated foods use in this study [13, 14] (Table 2); 50.67% of the RTE sesame seed are satisficing while 17.33% are acceptable and 32% are not satisficing. The discrepancies between the numbers of microorganisms of these three types of ready-to-eat sesame may reflect differences of food handler’s hygiene management and the final product storage. The flowchart of the RTE sesame processing (Fig. 4) showed clearly that no heating step is involved in the production of raw sesame seed. These seeds have just been cleaned (removal of physical matter), washed, dried and packed in a plastic bag under ambient conditions. This is what probably justifies its high load in microorganisms and which makes it one of the most contaminated and risky foods. Alaouie et al. [15] found out an average of 4.53 log_10_UFC.g^− 1^ (3.4 × 10^4^); 4.83 log_10_UFC.g^− 1^ (6.9 × 10^4^); 4.36 log_10_UFC.g^− 1^ (2.3 × 10^4^) respectively for coliforms, aerobic plate counts and yeasts and moulds while assessing the microbial quality of tahini (Sesame Paste) in Lebanon. In the other hand [16], found out an average of 1.44 log_10_UFC.g^− 1^ (2.8 × 10); 4.63 log_10_UFC.g^− 1^ (4.3 × 10^4^); 3.88 log_10_UFC.g^− 1^ (7.6 × 10^3^) respectively for coliforms, aerobic plate counts and yeasts and moulds while assessing the microbiological and chemical quality of tahini halva in Turkey. The obtained results in this study are slightly different from these two-research finding. Indeed, all these products are sesame-based products and the presence of microbes according to [15] has been attributed to a number of reasons including, the microbial quality of sesame seeds, poor hygiene and sanitation, and improper processing and storage conditions.

**Table 2:** Appreciation criteria of samples

	m (CFU/g)	M (CFU/g)
Total aerobic mesophilic bacteria ^a^	3.10^4^	3.10^5^
Thermotolerants coliforms ^a^	10^2^	10^3^
*E. coli* ^*a, b*^	10	10^2^
Yeasts and moulds ^a^	10^3^	10^4^
*Salmonella* ^*a, b*^	Absence /25 g	

^a, b^ Criteria (a = [13]; b = [14])

The international trade command sesame seed to be in a best quality as possible that mean free of bacteria such as *Salmonella* and other chemicals such as pesticides [17]. Sesame seed and sesame-based products export could be jeopardized if *Salmonella* was found. Published papers reported that sesame-based products such as Tahini and Halva (Helva) has been linked to *Salmonella* infections outbreak [16, 18–20]. In the recent years, a multi-country outbreak of new *Salmonella enterica* 11:z41:e,n,z15 infections associated with sesame seeds was been reported in Greece, Germany, Czech Republic, Luxembourg and United Kingdom [21]. The causes of these contaminations are mainly due to contaminated ingredients, poor quality of raw materials or unhygienic processing. It is believed that contamination of sesame-based products is deeply linked to the quality of the raw materials as sesame seed drying is done at open area. Sesame seed are thus exposed to all kinds of physical, chemical and biological contaminations. Most of the time, the soil, the manure, the feces of poultry and birds helped by the wind are responsible of microbial contamination [17]. exhibited 24.6% *Salmonella* contamination out of 359 raw untreated sesame seed samples submitted to exportation from 2007 to 2017 in Burkina Faso. According to [5], in Burkina Faso, sesame-based products processing is run by artisanal and semi-artisanal processors who are individuals, companies, associations and some pastries. Therefore, there is a need as stipulated by the [18] to design and to set up proper Hazard Analysis Control Critical Point (HACCP) system and Good Manufacturing Practices (GMP) as the contributing factors for reduction of microbial contamination in low-moisture foods.

Indeed, the processing methods used to transform raw sesame seed into dehulled sweet croquette and dehulled salted sesame seed should normally reduce drastically the microorganism population. We can hypothesize that the presence of microorganisms could then be linked to post contaminations or plastic bags used for conditioning. Our hypothesis was clearly confirmed by the presence of thermotolerant coliforms in 32 out of 75 samples (42.70%) including heat treated sesame seed. Most of them were found particularly in 89,33% of the raw sesame seed samples (17 out of 25). The presence of thermotolerants coliforms indicates the presence of fecal material from warm-blooded animals [22]. The thermotolerants coliforms (fecal coliforms) group is restricted to organisms that grow in the gastrointestinal tract of humans and other warm-blooded animals and includes members of at least 3 genera: *Escherichia, Klebsiella*, and *Enterobacter* [23]. The process used to obtain the three types of RTE sesame involved some critical points as highlighted in Fig. 4 by red color. These critical points could be the ways through which microorganisms could reach the final product and cause public health issue. Controlling these critical points is essential to improve the quality of the final product. During the processing of sweet croquette and salted dehulled sesame seed, a large amount of sugar and salt were added to give the sweet taste and dirt but also to improve the conservation of these products. Even thought, some sesame seed samples seem to have high number of microorganisms. According to [4], the addition of large amounts of salt or sugar can also be regarded as a simulated drying process, as it results in a reduction of the amount of water available for microbial growth. The dehulled sweet sesame croquette after caramelization undergoes a manual kneading operation in order to give the desired shapes (circles, heart, lozenge …) to the final products (Fig. 4). This could be one of the reasons for its high coliform load. Sanitation and personal hygiene, especially during home-based food processing, need improvement. According to [24], the tendency at present is to market dried fruits in the packaged form, the principal chances for contamination after drying would be from the hands of the person filling the package, if filling is done by hand, or from the package itself. Furthermore, there is a variation between the sesame seed samples in terms of visual quality. Some are more grilled, sweeter or more salted within the same group of samples. This suggested a lack of quality standards in sesame processing conditions. Therefore, it seems necessary to setup and respect Good Manufacturing practices (GMP), Good Hygiene Practices (GHP) and Good Standard Processing (GSP) by training the actors as the processing system of this field is still artisanal.

In general, survival, growth and multiplication of microorganisms in food depend on various factors which may be classified simply into those that are intrinsic or associated with the food material and those that are extrinsic or associated with the environment surrounding the food [25]. Therefore, all materials used for processing should be cleaned immediately in order to ensure the quality of the next production as the total aerobic mesophilic bacteria is high in almost all the sample (92%) and varies from 3 × 10 to 6.12 × 10^5^ CFU g^− 1^. Aerobic mesophilic bacteria are fermentative bacteria involved in foodshel degradation and contribute to lower the merchantable quality of food.

The presence of possible pathogenic organisms in these foodstuffs suggest a potential public health hazard to consumers. Fortunately, based on the present results, no sesame seed food item emerged as possible source of the infections, as neither *Salmonella spp* nor *E. coli* were found in any samples. These findings might reflect good hygienic practices of the sesame sample submitted to this research work. Torlak et al. [26] performed studies on matrices other than sesame seeds and concluded that the likely cause of the *Salmonella* outbreaks linked to sesame seed products was cross contamination of the products after the heat treatment. It was expected to find those pathogens in the raw samples as no heating step are involved in this process. Biochemical tests used to confirm suspected *E. coli* was found to be some *Klebsiella* and *Enterobacter* species. Further molecular investigations are deeply needed to understand why no *Salmonella* spp. neither *E. coli* were not found in these sesame seed samples even in high load of thermotolerants coliforms. According to [26] the standard roasting process is sufficient to inactivate *Salmonella* in sesame seeds. Indeed, raw sesame seed and sesame-based products are known to harbor sometimes *Salmonella* species. Previous studies on food quality in Burkina Faso raised the circulating pathogens such as *E. coli, Salmonella and Campylobacter*. Kagambèga et. al [27, 28] highlight the presence of *Salmonella enterica*, *Campylobacter* and *E. coli* in raw meat, poultry feces and carcasses in while [29] raised *E. coli* and *Salmonella* strains in milk and [30] isolated *E. coli* in Organic Waste Products from Cattle’s Markets. Foodborne illnesses in Burkina Faso have been liked to food chemical contamination. Recently in May 2013, in the Boulkiemdé province six persons of the same family die because of food intoxication (http://french.china.org.cn/foreign/txt/2021-05/23/content_77520820.htm consulted 28/05/2013). Fortunately, no illness has no yet been linked to sesame seed or sesame-based products.

Statistical analysis revealed that there is correlation between the three different parameters (*p*-value < 0.05) and the strength of this correlation, according to Pearson were 46.3%, 47.7% and 75.4% respectively between the total aerobic mesophilic bacteria and yeasts and moulds; between total aerobic mesophilic bacteria and the thermotolerant coliforms and finally between the thermotolerant coliforms and yeasts and moulds.

Sesame seeds are dehiscent dry food that low moisture allowed it to be stored at ambient temperature without any treatment. In the absence of sesame safety standards, weak conditioning systems can favor microbial growth to unacceptable levels even supplement such as sugar or salt have been added.

## Conclusion

RTE sesame seeds products are produced and sold everywhere in Burkina-Faso by women processors associations. This business provides them some revenues to fight poverty. However, to the  presence of high rate of microorganism in these street food sesames products which showed 32% of not satisficing not only point out the contamination rate due to intrinsic or extrinsic factors, but also highlight the high risk poses by these foods to consumers specifically in crowdy places. This manuscript, therefore, gives a snapshot of the hygienic level of some sesame sold in Burkina Faso but a more extensive survey would be required to find the level of contamination and if there is a public health concern. The contamination rate of the RTE sesame, processes and sol everywhere in Burkina Faso can lead to microorganism’s transportation. The importance of better organization of this field, adequate training for RTE sesame handlers and their managers; in the view to decrease contamination rate is therefore emphasized.

## Data Availability

The datasets used and/or analyzed during the current study are available from the corresponding author on reasonable request.

## Abbreviations

RTE: Ready-To-Eat
CFU: Colony forming unit
IMViC: Indole, Methyl red, Vogues-Proskauer, Citrate
DW: Sweet dehulled sesame croquettes
DS: Salted dehulled sesame seed
RS: Raw sesame seed
GMP: Good manufacturing practices
GHP: Good hygiene practices
GSP: Good standard processing
CDC: Centers for Disease Control and Prevention
